# Teledentistry actions in Primary Healthcare during the COVID-19 pandemic: cross-sectional study, Brazil, 2021

**DOI:** 10.1590/S2237-96222025v34e20240669.en

**Published:** 2025-09-08

**Authors:** Mariane Baltassare Laroque, Thais Freitas Formozo Tillmann, Bruna Vettorazzi Liskoski, Alexandre Emidio Ribeiro Silva

**Affiliations:** 1 Universidade Federal de Pelotas, Pelotas, Programa de Pós-Graduação em Odontologia, Pelotas, RS, Brazil; 2 Universidade Federal de Pelotas, Graduação em Odontologia, Pelotas, RS, Brazil

**Keywords:** COVID-19, Primary Healthcare, Teledentistry, Public Health, Cross-Sectional Studies, Covid-19, Atención Primaria de Salud, Teleodontología, Salud Pública, Estudios Transversales

## Abstract

**Objective:**

To analyze the use of teledentistry in Primary Healthcare in Brazil at the end of the second year of the COVID-19 pandemic.

**Methods:**

Cross-sectional study with dentists and dental surgeons in Primary Healthcare. Study data were obtained through an online form. The outcome was the use of teledentistry in Basic Health Units. The exposure variables were related to dentists, municipalities and the adoption of protocols by the municipality to reduce the risk of COVID-19. Descriptive and bivariate analyses were performed, and finally, adjusted regression analysis was performed, in which prevalence ratios (PR) and 95% confidence intervals (95%CI) were obtained.

**Results:**

416 dentists were evaluated. Of these, 24.3% reported using teledentistry, with remote guidance being the most used modality (85.6%). The highest probability of implementing teledentistry occurred in Basic Health Units in the South (RP 4.20; 95%CI 1.71; 10.28) and Southeast (RP 6.85; 95%CI 2.74; 17.67) regions of Brazil, which adopted protocols to reduce the risk of COVID-19 (PR 1.59; 95%CI 1.01; 2.50) and had dentists specialized in Public/Collective Health (PR 2.12; 95%CI 1.20; 3.75).

**Conclusion:**

Limited use of teledentistry was observed by Basic Health Units in Brazil. Factors related to the adoption of protocols to reduce the risk of COVID-19, the Brazilian region of the Basic Health Unit and the presence of a dentist with specialization in Public/Collective Health were positively associated with the implementation of teledentistry.

## Resumen

**Objetivo:**

Analizar el uso de la teleodontología en la Atención Primaria de Salud en Brasil al final del segundo año de la pandemia de covid-19.

**Métodos:**

Estudio transversal con odontólogos de atención primaria de salud. Los datos del estudio se obtuvieron a través de un formulario en línea. El resultado fue la utilización de la teleodontología en las Unidades Básicas de Salud. Las variables de exposición se relacionaron con los dentistas, los municipios y la adopción de protocolos por parte del municipio para reducir el riesgo de covid-19. Se realizaron análisis descriptivos y bivariados, y finalmente, análisis de regresión ajustado, en el que se obtuvieron razones de prevalencia (RP) e intervalos de confianza del 95% (IC95%).

**Resultados:**

Se evaluaron 416 odontólogos. De estos, el 24,3% reportó utilizar teleodontología, siendo la teleguía la modalidad más utilizada (85,6%). La mayor probabilidad de implementación de la teleodontología ocurrió en las Unidades Básicas de Salud de las regiones Sur (RP 4,20; IC95% 1,71; 10,28) y Sudeste (RP 6,85; IC95% 2,74; 17,67) de Brasil, que adoptaron protocolos para reducir el riesgo de covid-19 (RP 1,59; IC95% 1,01; 2,50) y contaban con dentistas especializados en Salud Pública/Colectiva (RP 2,12. IC95% 1,20; 3,75).

**Conclusión:**

Hubo uso limitado de la teleodontología en las Unidades Básicas de Salud en Brasil. Los factores relacionados con la adopción de protocolos para reducir el riesgo de covid-19, la región brasileña de la Unidad Básica de Salud y la presencia de un dentista con especialización en Salud Pública/Colectiva se asociaron positivamente con la implementación de la teleodontología.

**Ethical aspects**

This research respected ethical principles, having obtained the following approval data:

Research Ethics Committee: Universidade Federal de Pelotas

Opinion number: 33837220.4.00005317

Approval date: 29/6/2020

Informed Consent Form: Obtained from all participants prior to collection of samples.

## Introduction

Dentistry has been greatly impacted by the emergence of COVID-19 (1), and this is considered a high-risk profession for contagion and dissemination of the SARS-CoV-2 virus through aerosols (2). In view of this situation, biosafety protocols were published to ensure greater safety for dentists and users of dental services (3).

The adoption of measures to minimize cases of infection among professionals and users directly affected Primary Healthcare services, which are responsible for providing dental care to a large part of the Brazilian population (4). A significant reduction in dental procedures was also observed in Secondary Healthcare services, where endodontics and periodontics specialties were strongly impacted (5).

To improve the quality of care, expand the actions offered by health teams and, in times of pandemic, reduce contamination by the SARS-CoV-2 virus, the use of telehealth is an information and communication strategy for exchanging health data and information (6,7). In Dentistry, teledentistry emerges as a field of telehealth, developing actions such as remote guidance, telemonitoring, teleinterconsultation and teletriage (8).

The COVID-19 pandemic has boosted the incorporation of teledentistry, significantly increasing knowledge about this technology and its practice (9). In Brazil, the Federal Council of Dentistry, which regulates the profession of dentists, published Resolution 226/2020 in June 2020, which regulated the use of teledentistry (3). The Brazilian Dental Education Association reiterated this position, publishing some guidelines aimed at higher education institutions in the dental field, highlighting its great usefulness and recommending that these entities take ownership of the discussions and advances achieved. in this area (10).

This recent technology has proven to be quite useful in minimizing patient visits to the dental clinic during lockdown times (11), proving to be important for carrying out screenings, consultations, diagnoses and monitoring of patients, as well as for prescribing medication, including antimicrobials (12). Therefore, the present study aims to analyze the use of teledentistry in Primary Healthcare in Brazil, at the end of the second year of the COVID-19 pandemic. 

## Methods

### Study design

This is a cross-sectional study with dentists working in Primary Healthcare services in Basic Health Units in Brazil during the COVID-19 pandemic. 

### Context 

Since the start of the pandemic in 2020, study researchers have conducted two follow-ups: a baseline study in 2020 and a first follow-up in 2021. The data evaluated in this study refers to the first follow-up.

Data collection for the first monitoring took place between November and December 2021, when COVID-19 vaccination coverage in Brazil had already exceeded 60.0% of the population with two doses of the vaccine. 

### Study participants 

All dentists who worked in Primary Healthcare services in Brazil in July 2020 were eligible to participate in the baseline study. At the time of the baseline study, according to records from the Primary Healthcare Department of the Brazilian Ministry of Health, there were approximately 20,000 dentists working in Primary Healthcare services. An email was sent to dentists explaining the objectives of the study and containing a link to the self-administered online questionnaire hosted on the Google Forms platform. In addition, publications were made through the studys social networks (Instagram) requesting the participation of dentists who were working in Primary Healthcare. At the end of the baseline study, in 2020, 947 dentists were evaluated. Of these, 720 reported that they were willing to participate in the first monitoring in 2021.

**Figure 1:**
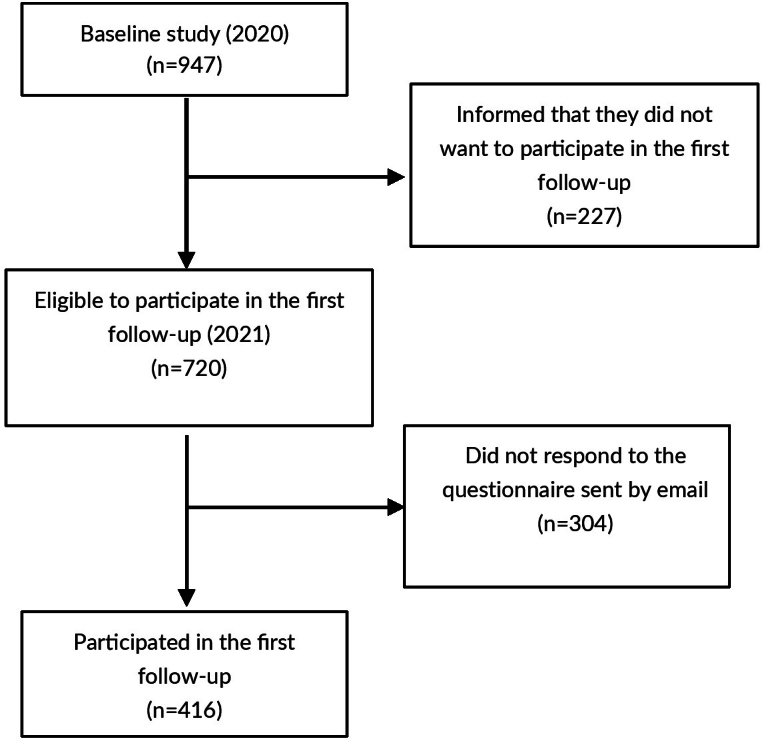
Flowchart of follow-ups with dentists working in Basic Health Units, at the end of the second year of the COVID-19 pandemic. Brazil, 2021

Only dentists who participated in the baseline study and who agreed to participate in new studies sending their contact email at the completion of the baseline study questionnaire were eligible to participate in the first follow-up in 2021. The exclusion criteria for the first follow-up were: not having participated in the baseline study; not having sent the email at the end of the baseline study agreeing to participate in the first follow-up and no longer being linked to Primary Healthcare services at the time of study data collection. Figure 1 shows the flowchart of the monitoring conducted. 

### Study variables 

The outcome analyzed in this study was the use of teledentistry in Basic Health Units at the end of the second year of the COVID-19 pandemic, obtained by the question: “Did your Basic Health Unit implement any form of Teledentistry during this period?” With the following answer options: Yes, No and I do not know. 

The following exposure variables related to dentists and the municipality in which they worked in Primary Healthcare were analyzed: sex (male or female); specialization in Public or Collective Health (No or Yes); Brazilian regions of Basic Health Units (Southeast; South; Midwest, North and Northeast) and adoption of protocols by the municipality to reduce the risk of COVID-19 (No or Yes). Furthermore, the modality of teledentistry implemented by the Basic Health Unit (remote guidance; telemonitoring and teleconsultation) and the dentist’s perception regarding the continued use of teledentistry after the pandemic (Yes or No) were verified. 

The technologies used were categorized according to the literature (13) as follows:

- Dental teleconsultation: communication between dental professionals using digital technologies, with the aim of exchanging information and opinion on diagnosis and clinical conduct;
- Remote guidance: identification and classification of health conditions, mediated by digital technologies;
- Telemonitoring: use of digital technologies for monitoring patients by the dentist after dental care, with the aim of monitoring their health status.

### Data Source/measurement 

For data collection, a self-administered online questionnaire was organized, hosted on the Google Forms platform. The collection instrument consisted of 7 blocks with sociodemographic questions, organization of services at Basic Health Units (infrastructure and routine of care), teledentistry and mental health. The full questionnaire is available as supplementary material. Contact with study participants was by email sent to 720 dentists who agreed to participate in other follow-ups, as indicated in the baseline study. An email was sent with the questionnaire link and the Informed Consent Formulary - ICF. To complete the questionnaire, the dentist had to read and agree to the ICF. To reach the largest number of participants, three attempts were made by email, over three consecutive weeks. When no response was received or the email address was not valid, the researchers also tried to locate the participants via social media. 

### Procedures to avoid potential sources of bias

Before collecting data for the first follow-up, a pilot study was carried out, in which the questions in the collection instrument were tested by 10 dentists who had passed a public exam in Primary Healthcare but who, at the time of the research, were working in dental specialty centers. Participants were asked to assess the clarity of the questions, record the total time taken to complete the questionnaire, and point out issues that might need to be reviewed by the study researchers. 

### Study size

The sample of the present study was non-probabilistic for convenience. 

### Statistical methods

Stata 12.0 software was used for statistical analysis of the data. Descriptive analyses were conducted using relative and absolute frequencies and averages. Subsequently, univariate analyses were performed using Fisher’s exact and chi-square tests. Finally, multivariate analysis, using Poisson regression and prevalence ratios (PR) and 95% confidence intervals (95%CI) were obtained. All variables with a p-value less than or equal to 0.2 were kept in the model for adjustment purposes. For all analyses, a significance level of 5.0% was considered. 

The study hypothesis is that less than 50.0% of dentists would have performed teledentistry actions in Primary Healthcare in Brazil at the end of the second year of the COVID-19 pandemic. 

## Results

416 dentists who were working in Primary Healthcare services in Brazil participated in the study. Most participants were female (74.5%) and worked in Basic Health Units located in the Southern region of Brazil (54.1%). Regarding professional training, 27.0% of dentists had graduated five years or less before the study, while 23.9% stated that they had graduated 21 years or more before the study. Of the 319 dentists who had postgraduate degrees at a specialization level, 74.6% were specialists in Public or Collective Health (Table 1). 

**Table 1:** Sociodemographic characteristics of dentists working in Basic Health Units and organization of Basic Health Units in Brazil, at the end of the second year of the COVID-19 pandemic. Brazil, 2021 (n=416)

Variables	n (%)
**Gender**	
Male	106 (25.5)
Female	310 (74.5)
**Time since graduating in dentistry** (years)^a^	
0 -5	112 (27.0)
6 -10	86 (20.7)
11-15	64 (15.4)
16-20	54 (13.0)
≥21	99 (23.9)
**Specialization in Public or Collective Health**	
No	81 (25.4)
Yes	238 (74.6)
**Region of the Basic Health Unit in Brazil**	
Southeast	83 (20.0)
South	225 (54.1)
Midwest	20 (4.8)
North and Northeast	83 (21.1)
**Adoption of a protocol by the municipality of the Basic Health Unit to reduce the risk of COVID**-19	
No	137 (32.9)
Yes	279 (67.1)
**Implementation of some teledentistry tool by the Basic Health Unit**	
No	315 (75.7)
Yes	101 (24.3)
**Intends to use Teledentistry after the pandemic^a^**	
No	60 (66.7)
Yes	30 (33.3)

^a^Variables with missing data.

**Figure 2:**
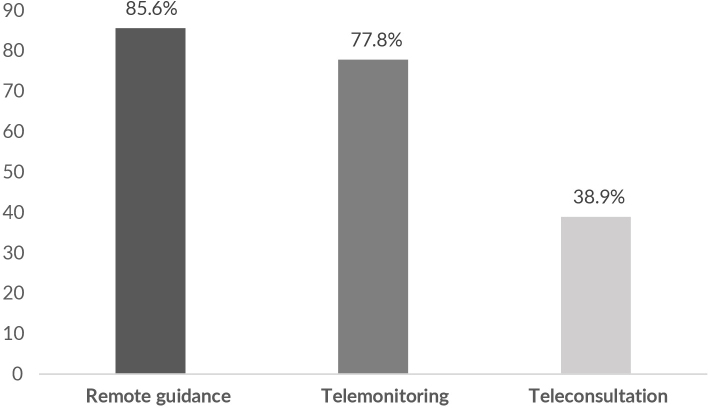
Percentage of teledentistry modalities used by dentists working in Basic Health Units in Brazil at the end of the second year of the COVID-19 pandemic. Brazil, 2021 (n=90)

The adoption of protocols to reduce the risk of COVID-19 by the municipality was reported by most dentists (67.1%). The adoption of some teledentistry tool during this period was reported by 24.3% of dentists. Of those who reported this, the most used teledentistry modality was remote guidance (85.6%) (Figure 2). Among those who declared the implementation of this technology, only 33.3% believed that it will continue to be used after the pandemic. 

Table 2 presents the results of the univariate analysis. Comparing the exposure variables with the study outcome, positive associations were observed between the implementation of teledentistry and Basic Health Units with female dentists (p-value 0.049), which had professionals with specialization in Public or Collective Health (p-value 0.002), that were located in the Southern region of Brazil (p-value 0.002) and the Basic Health Units that adopted some protocol to reduce the risk of COVID-19 (p-value<0.001).

**Table 2:** Comparison of the study’s exposure variables with the implementation of teledentistry in Primary Healthcare in Brazil at the end of the second year of the COVID-19 pandemic. Brazil, 2021 (n=416)

Variables	Implementation of teledentistry		
	No	Yes	
	n (%)	n (%)	p-value
**Sex** (n=416)			0.049
Male	88 (83.0)	18 (17.0) 83 (26.8)	
Female	227 (73.2)		
**Time since graduating in dentistry** (years) (n=415)^a^			0.429
0-5	89 (80.2) 67 (78.8) 48 (75.0) 38 (70.4) 70 (70.7)	22 (19.8) 18 (21.2) 16 (25.0) 16 (29.6) 29 (29.3)	
6-10			
11-15			
16-20			
≥21			
**Specialization in Public or Collective Health** (n=319)			0.002
No	70 (86.4) 164 (68.9)	11 (13.6) 74 (31.1)	
Yes			
**Region of the Basic Health Unit in Brazil** (n=416)			<0.001
Southeast	48 (57.8)	35 (42.2)	
South	168 (74.7)	57 (25.3)	
Midwest	16 (80.0)	4 (20.0)	
North and Northeast	83 (94.3)	5 (5.7)	
**Adoption of protocol by the municipality of the Basic Unit** **of Healthcare to reduce the risk of COVID-19** (n=416)			<0.001
No	119 (86.9)	18 (13.1)	
Yes	196 (70.3)	83 (29.7)	

^a^Variables with missing data.

Table 3 presents the results of the raw and adjusted multivariate analyses. After the raw analysis, the exposure variables that remained positively associated with the implementation of teledentistry were: the Brazilian region of the Basic Health Unit, adoption of a protocol by the municipality of the Basic Health Unit to reduce the risk of COVID-19 and the Basic Health Units where there were dentists with specialization in Public or Collective Health. In the adjusted analysis, these same variables also remained positively associated with the outcome. The location of the Basic Health Unit in the South and Southeast regions presented a 320% (PR 4.20; 95%CI 1.71; 10.28) and 585.0% higher probability (PR 6.85; 95%CI 2.74; 17.67), respectively, of implementing teledentistry when compared to the North and Northeast regions. Furthermore, those dentists who reported the adoption of a protocol by the municipality of the Basic Health Unit to reduce the risk of COVID-19 were 59.0% more likely to implement teledentistry compared to those who did not implement it (PR 1.59; 95%CI 1.01; 2.50). The Basic Health Units that had dentists specialized in Public or Collective Health, there was a 112.0% greater probability of implementing teledentistry compared to those Basic Health Units that did not have these professionals (PR 2.12; CI95% 1.20; 3.75). 

**Table 3:** Crude and adjusted prevalence ratios (PR) and 95% confidence intervals (95%CI) of the implementation of teledentistry according to the study variables at the end of the second year of the COVID-19 pandemic. Brazil, 2021 (n=416)

	Implementation of teledentistry					
	Raw analysis			Adjusted analysis		
Variables	PR	(95%CI)	p-value	PR	(95%CI)	p-value
**Gender**			0.052	-		
Male	1.00					
Female	1.57	(0.99; 2.49)				
**Time since graduating in dentistry** (**^y^ears)a**			0.435	-		
0-5	1.00					
6-10	1.06	(0.61; 1.86)				
11-15	1.26	(0.71; 2.22)				
16-20	1.49	(0.85; 2.60)				
≥21	1.47	(0.91; 2.39)				
**Specialization in Public or Collective Health**			0.005			0.010
No	1.00			1.00		
Yes	2.29	(1.27; 4.09)		2.12	(1.20; 3.75)	
**Region of the Basic Health Unit in Brazil**			0.001			0.001
North and Northeast	1.00			1.00		
Midwest	3.52	(1.03; 11.96)		3.09	(0.87; 10.93)	
South	4.45	(1.84; 10.76)		4.20	(1.71; 10.28)	
Southeast	7.42	(3.05; 18.05)		6.85	(2.74; 17.67)	
**Adoption of protocol by the municipality of the Basic Health Unit to reduce the risk of COVID-19**			0.001			0.046
No	1.00			1.00		
Yes	2.26	( 1.41; 3.61)		1.59	(1.01; 2.50)	

^a^Variables with missing data.

## Discussion

The main characteristic of this study was to identify aspects related to the use of teledentistry in Brazil during the COVID-19 pandemic. The results showed that, although protocols to reduce the risk of COVID-19 were implemented in most of the municipalities where the dentists involved in the study worked, teledentistry tools were used in only a quarter of the municipalities, confirming the study hypothesis. Remote guidance was the most used form of teledentistry, according to participants. Furthermore, factors such as the location of the Basic Health Unit in the South and Southeast regions, and the fact that dentists are specialists in Public or Collective Health favored the use of this tool. The authors of this study believe that the results obtained, in a context of health crisis, highlight the importance of including teledentistry modalities in the routine of Primary Healthcare teams, promoting greater prevention and control of problems related to oral health for the entire population. To the authors’ knowledge, there are no publications on teledentistry actions in Primary Healthcare in Brazil during the COVID-19 pandemic. 

The adoption of municipal protocols to reduce the risk of COVID-19 remained positively associated with the study outcome. It is important to highlight that all biosafety measures for clinical management were essential during the pandemic, given that dentists had direct contact with aerosols that could be contaminated by the virus. Therefore, protocols had to be adopted from the patient’s arrival until they left the office, including measures to reduce waiting time and clinical management, aiming to minimize contact between the patient and the professional (14). A study that analyzed the factors associated with the reduction in the number of dental appointments performed in Primary Healthcare in Brazil in the first year of the pandemic noted that 67.1% of municipalities adopted protocols to reduce the risk of contamination by the virus, and 29.7% implemented some type of screening (such as temperature measurement, identification of signs and symptoms of COVID-19, among others) in the Basic Health Unit (15). The adoption of new biosafety protocols, including teletriage, may have been an important factor for the implementation of telemonitoring and remote guidance actions observed in the present study, especially for users who were receiving care in health units before the start of the pandemic, as most municipalities in Brazil only provided urgent and emergency care during the first year of the pandemic (3). 

Basic Health Units with dentists specializing in Public or Collective Health implemented more teledentistry tools compared to Basic Health Units that did not have these professionals. For the proper functioning of the health unit and the development of teledentistry actions, it is essential that human resources are trained to use the technologies. Health professionals specializing in Public or Collective Health are trained to recognize the importance and need for continuous improvement of health services provided to the population. The presence of well-structured and organized services, combined with professionals committed to their proper functioning, promotes positive impacts on the health of the population and increases the efficiency of the system (16).

Basic Health Units located in the South and Southeast regions implemented more teledentistry actions compared to the North and Northeast regions. Until August 2024, the Southern region of Brazil offered the Telessaúde RS service, a Telemedicine initiative of the Brazilian Ministry of Health in collaboration with the Federal University of Rio Grande do Sul, since 2007, with the objective of promoting teleconsultation and tele-education actions for Primary Healthcare professionals (17). This fact may have contributed to the results found in the present study, since the implementation of teledentistry actions requires an adequate technical and network infrastructure, with adequate internet connection, in addition to the need for team training. It takes time for professionals to adapt to the system so that the service can be used efficiently (18,19). 

The study collected data from all regions of Brazil. However, the sample was not representative of the entire country, as most of the study participants were from the southern region of the country (around 50.0%), which can be considered a limitation of the study. It is worth noting, however, that the researchers tried to contact dentists eligible to participate, using emails and social networks. It is important to highlight that, due to the pandemic, online research was the only way to learn and understand how oral health services in Primary Healthcare were organized to face the public health emergency. One factor that can contribute to strengthening teledentistry actions in the post-pandemic period are the efforts of the Secretariat of Information and Digital Health of the Ministry of Health in proposing strategies related to digital health for the period 2020-2028, through the National Health Data Network. Among these initiatives, the creation of a digital platform for innovation, information and health services stands out, with actions aimed at monitoring and evaluating digital health throughout Brazil (20). 

Finally, considering the objective of this study, we can state that the adoption of protocols to reduce the risk of COVID-19, the specific Brazilian region of the Basic Health Unit and the presence of a dentist with specialization in Public or Collective Health were positively associated with the implementation of teledentistry. Furthermore, a limited use of teledentistry tools was observed in Brazilian Basic Health Units at the end of the second year of the COVID-19 pandemic. These results serve as a warning that actions related to teledentistry, especially those aimed at monitoring and teleconsulting, should be implemented continuously and integrated into the National Oral Health Policy, not only in times of health crisis, but as a permanent strategy to improve oral health indicators and, consequently, the population’s quality of life.

## Data Availability

After publication, the data will be available on demand by contacting the authors.
