# Supplementary material for: Teledentistry actions in Primary Healthcare during the COVID-19 pandemic: cross-sectional study, Brazil, 2021
Source: Epidemiol Serv Saude. 2025 Sep 8;34:e20240669. doi: 10.1590/S2237-96222025v34e20240669.en (PMC12435950; doi:10.1590/S2237-96222025v34e20240669.en)
Supplement: Translation

# Translation: Ações da teleodontologia na Atenção Primária à Saúde durante a pandemia de covid-19: estudo transversal, Brasil, 2021

**DOI:** 10.1590/S2237-96222025v34e20240669.pt

**Authors:** Mariane Baltassare Laroque, Thais Freitas Formozo Tillmann, Bruna Vettorazzi Liskoski, Alexandre Emidio Ribeiro Silva

**Affiliations:** 1 Universidade Federal de Pelotas, Pelotas, Programa de Pós-Graduação em Odontologia, Pelotas, RS, Brasil; 2 Universidade Federal de Pelotas, Graduação em Odontologia, Pelotas, RS, Brasil

**Keywords:** Covid-19, Atenção Primária à Saúde, Teleodontologia, Saúde Pública, Estudos Transversais

## Abstract

**Objetivo:**

Analisar a utilização da teleodontologia na Atenção Primária à Saúde no Brasil ao final do segundo ano da pandemia da covid-19.

**Métodos:**

Estudo Transversal com cirurgiões-dentistas da Atenção Primária à Saúde. Os dados do estudo foram obtidos por meio de um formulário online. O desfecho foi a utilização da teleodontologia nas Unidades Básicas de Saúde. As variáveis de exposição estavam relacionadas aos cirurgiões-dentistas, aos municípios e à adoção de protocolos pelo município para reduzir risco da covid-19. Foram realizadas as análises descritivas, bivariadas e por fim, a análise de regressão ajustada na qual foram obtidas as razões de prevalência (RP) e intervalos de confiança de 95% (IC95%).

**Resultados:**

Foram avaliados 416 cirurgiões-dentistas. Destes, 24,3%, relataram fazer uso da teleodontologia, sendo a teleorientação a modalidade mais utilizada (85,6%). A maior probabilidade de implementar a teleodontologia ocorreu nas Unidades Básicas de Saúde das regiões Sul (RP 4,20; IC95% 1,71; 10,28) e Sudeste (RP 6,85; IC95% 2,74; 17,67) do Brasil, que adotaram protocolos para a redução do risco da covid-19 (RP 1,59; IC95% 1,01; 2,50) e contavam com cirurgiões-dentistas especialistas em Saúde Pública/Coletiva (RP 2,12; IC95% 1,20; 3,75).

**Conclusão:**

Observou-se um limitado uso da teleodontologia pelas Unidades Básicas de Saúde do Brasil. Fatores relacionados à adoção de protocolos para redução de risco da covid-19, a região brasileira da Unidade Básica de Saúde e a presença de um cirurgião-dentista com especialização em Saúde Pública/Coletiva, foram associadas positivamente à implementação da teleodontologia.

**Aspectos éticos**

A presente pesquisa respeitou os princípios éticos, obtendo os seguintes dados de aprovação:

Comitê de ética em pesquisa: Universidade Federal de Pelotas

Número do parecer: 33837220.4.00005317

Data de aprovação: 29/6/2020

Registro do consentimento livre e esclarecido: Obtido de todos os participantes antes da coleta.

## Introdução

A Odontologia sofreu um grande impacto a partir do surgimento da covid-19 (1), sendo considerada uma profissão de alto risco para contágio e disseminação do vírus Sars-CoV-2 por meio de aerossóis (2). Diante dessa situação, foram publicados protocolos de biossegurança para garantir maior segurança aos cirurgiões-dentistas e para os usuários dos serviços odontológicos (3).

A adoção de medidas para minimizar ao máximo o número de casos de infecções em profissionais e usuários afetou diretamente os serviços de Atenção Primária à Saúde, que é responsável pelo atendimento odontológico de uma grande parte da população do Brasil (4). Uma redução significativa de procedimentos odontológicos também foi observada nos serviços de Atenção Secundária, onde as especialidades de endodontia e periodontia foram fortemente impactadas (5).

Para melhorar a qualidade do atendimento, ampliar as ações ofertadas pelas equipes de saúde e, em momentos de pandemia, reduzir a contaminação pelo vírus Sars-CoV-2, o uso da telessaúde, é uma estratégia de informação e comunicação para troca de dados e informações de saúde (6,7). Na Odontologia, a teleodontologia surge como um campo da telessaúde, desenvolvendo ações como a teleorientação, telemonitoramento, teleinterconsulta e teletriagem (8).

A pandemia da covid-19 impulsionou a incorporação da teleodontologia, aumentando de maneira significativa o conhecimento sobre essa tecnologia e sua prática (9). No Brasil, o Conselho Federal de Odontologia, que regula a profissão do cirurgião-dentista, publicou em junho de 2020, a Resolução 226/2020, que regulamentou a utilização da teleodontologia (3). A Associação Brasileira de Ensino Odontológico reiterou esse posicionamento, publicando algumas orientações voltadas para as instituições de ensino superior na área odontológica, destacando a sua grande utilidade e recomendando que essas entidades se apropriem das discussões e dos avanços conquistados nessa área (10).

Essa nova tecnologia provou ser bastante útil ao minimizar as visitas dos pacientes à clínica odontológica em tempos de lockdown (11), mostrando-se importante para a realização de triagens, consultas, diagnósticos e monitoramento de pacientes, bem como para a prescrição medicamentosa, inclusive de antimicrobianos (12). Portanto, o presente trabalho tem por objetivo analisar a utilização da teleodontologia na Atenção Primária à Saúde, no Brasil, ao final do segundo ano da pandemia da covid-19. 

## Métodos

### Delineamento do estudo

Este é um estudo transversal com cirurgiões-dentistas atuantes nos serviços de Atenção Primária à Saúde em Unidades Básicas de Saúde do Brasil durante a pandemia da covid-19. 

### Contexto 

Desde o início da pandemia, em 2020, os pesquisadores do estudo realizaram dois acompanhamentos: estudo de base em 2020 e primeiro acompanhamento em 2021. Os dados avaliados neste estudo referem-se ao primeiro acompanhamento.

A coleta de dados do primeiro acompanhamento ocorreu entre novembro e dezembro de 2021 quando a cobertura vacinal da covid-19 no Brasil já superava 60,0% da população com duas doses da vacina. 

### Participantes do estudo 

Eram elegíveis para participar do estudo de base todos os cirurgiões-dentistas que estivessem vinculados aos serviços de Atenção Primária à Saúde no Brasil em julho de 2020. No momento do estudo de base, conforme os registros do Departamento de Atenção Primária à Saúde do Ministério da Saúde, existiam aproximadamente 20 mil dentistas atuando nos serviços de Atenção Primária. Um e-mail foi enviado aos cirurgiões-dentistas explicando os objetivos do estudo e contendo um link para o questionário autoaplicável online hospedado na plataforma Google Forms. Além disso, foram realizadas publicações por meio das redes sociais do estudo (Instagram) solicitando a participação dos cirurgiões-dentistas que estivessem atuando na Atenção Primária à Saúde. Ao final do estudo de base, em 2020, foram avaliados 947 cirurgiões-dentistas. Desses, 720 informaram que poderiam participar do primeiro acompanhamento em 2021.

Somente os cirurgiões dentistas que participaram do estudo de base e que tenham autorizado a participação em novos estudos, enviando o seu e-mail de contato ao final do preenchimento do questionário do estudo de base, estavam aptos a participar do primeiro acompanhamento em 2021. Os critérios de exclusão do primeiro acompanhamento foram: não ter participado do estudo de base; não ter enviado o e-mail ao final do estudo de base autorizando a participação no primeiro acompanhamento, e não estar mais vinculado aos serviços de Atenção Primária à Saúde no momento da coleta de dados do estudo. A figura 1 apresenta o fluxograma dos acompanhamentos realizados. 

**Figura 1:** Fluxograma dos acompanhamentos com cirurgiões-dentistas atuantes nas Unidades Básicas de Saúde, ao final do segundo ano da pandemia de covid-19. Brasil, 2021

### Variáveis do estudo 

O desfecho analisado no presente estudo foi a utilização da teleodontologia nas Unidades Básicas de Saúde ao final do segundo ano da pandemia da covid-19, obtido pela pergunta: “A sua Unidade Básica de Saúde instituiu alguma forma de Teleodontologia nesse período?” Com as seguintes opções de resposta: sim, não e não sei. 

Foram analisadas as seguintes variáveis de exposição relacionadas aos cirurgiões-dentistas e ao município em que atuavam na Atenção Primária à Saúde: sexo (masculino ou feminino); especialização em Saúde Pública ou Coletiva (não ou sim); regiões brasileiras das Unidades Básicas de Saúde (Sudeste; Sul; Centro-oeste, Norte e Nordeste) e adoção de protocolos pelo município para reduzir risco da covid-19 (não ou sim). Além disso, foi verificada qual a modalidade de teleodontologia implementada pela Unidade Básica de Saúde (teleorientação; telemonitoramento e teleinterconsulta) e a percepção do cirurgião-dentista sobre a continuidade da utilização da teleodontologia após a pandemia (não ou sim). 

As tecnologias utilizadas foram categorizadas de acordo com a literatura (13) da seguinte forma:

- Teleconsultoria odontológica: comunicação entre profissionais da odontologia, utilizando tecnologias digitais, com o objetivo de trocar informações e opiniões sobre diagnóstico e conduta clínica;
- Teleorientação: identificação e classificação de condições de saúde, mediadas por tecnologias digitais;
- Telemonitoramento: uso de tecnologias digitais para acompanhamento de pacientes pelo cirurgião-dentista após atendimento odontológico, com o objetivo de monitorar o estado de saúde.

### Fonte de dados/mensuração 

Para a coleta de dados, foi organizado um questionário autoaplicável online, hospedado na plataforma Google Forms. O instrumento de coleta era composto por 7 blocos com questões sociodemográficas, de organização dos serviços das Unidades Básicas de Saúde (infraestrutura e rotina dos atendimentos), teleodontologia e saúde mental. O questionário completo está disponível como material suplementar. O contato com os participantes do estudo foi por meio do envio de um e-mail aos 720 cirurgiões-dentistas que aceitaram participar de outros acompanhamentos, conforme indicado no estudo de base. Um e-mail foi enviado com o link do questionário e o termo de consentimento livre e esclarecido - TCLE. Para preencher o questionário, o cirurgião dentista tinha que ler e concordar com o TCLE. Para alcançar o maior número de participantes foram feitas três tentativas por e-mail, durante três semanas consecutivas. Quando não se obtinha resposta ou o endereço de e-mail não era válido, os pesquisados também tentaram localizar os participantes via redes sociais. 

### Procedimentos para contornar potenciais fontes de viés

Antes da coleta dos dados do primeiro acompanhamento, realizou-se um estudo piloto, no qual as questões do instrumento de coleta foram testadas por 10 cirurgiões-dentistas concursados na Atenção Primária à Saúde, mas que, no momento da pesquisa, atuavam nos centros de especialidades odontológicas. Solicitou-se aos participantes que avaliassem a clareza das perguntas, registrassem o tempo total para o preenchimento do questionário e apontassem questões que, porventura devessem ser revistas pelos pesquisadores do estudo. 

### Tamanho do estudo

Amostra do presente estudo foi não probabilística por conveniência. 

### Métodos estatísticos

Para a análise estatística dos dados foi utilizado o software Stata 12.0. Foram realizadas análises descritivas, por meio frequências relativas e absolutas e médias. Posteriormente, foram realizadas análises univariadas utilizando os testes Exato de Fisher e Qui-quadrado. Por fim, a análise multivariada, por meio da regressão de Poisson e obtidas as razões de prevalência (RP) e intervalos de confiança de 95% (IC95%). Foram mantidas no modelo, para fins de ajuste, todas as variáveis com p-valor menor ou igual a 0,2. Para todas as análises, foi considerado um nível de significância de 5,0%. 

A hipótese do estudo é que menos de 50.0% dos cirurgiões-dentistas teriam realizado ações de teleodontologia na Atenção Primária à Saúde no Brasil ao final do segundo ano da pandemia da covid-19. 

## Resultados

Participaram do estudo 416 cirurgiões-dentistas que estavam atuando nos serviços de Atenção Primária à Saúde do Brasil. A maioria dos participantes era do sexo feminino (74,5%) e trabalhava em Unidades Básicas de Saúde localizadas na região Sul do Brasil (54,1%). Em relação à formação profissional, 27,0% dos cirurgiões-dentistas tinham, no máximo, cinco anos de formado, enquanto 23,9% declararam ter 21 anos ou mais de formado. Dos 319 cirurgiões-dentistas que tinham pós-graduação em nível de especialização, 74,6% eram especialistas em Saúde Pública ou Coletiva (Tabela 1). 

**Tabela 1:** Características sociodemográficas dos cirurgiões-dentistas atuantes nas Unidades Básicas de Saúde e organização das Unidades Básicas de Saúde do Brasil, ao final do segundo ano da pandemia de covid-19. Brasil, 2021 (n=416)

Variáveis	n (%)
**Sexo**	
Masculino	106 (25,5)
Feminino	310 (74,5)
**Tempo de formado em odontologia** (anos)^a^	
0-5	112 (27,0)
6-10	86 (20,7)
11-15	64 (15,4)
16-20	54 (13,0)
≥21	99 (23,9)
**Especialização em Saúde Pública ou Coletiva**	
Não	81 (25,4)
Sim	238 (74,6)
**Região do Brasil da Unidade Básica de Saúde**	
Sudeste	83 (20,0)
Sul	225 (54,1)
Centro-Oeste	20 (4,8)
Norte e Nordeste	83 (21,1)
Adoção de protocolo pelo município da Unidade Básica de Saúde para reduzir risco de covid-19	
Não	137 (32,9)
Sim	279 (67,1)
**Implementação de alguma ferramenta de teleodontologia pela Unidade Básica de Saúde**	
Não	315 (75,7)
Sim	101 (24,3)
**Teleodontologia permanecerá sendo utilizada após pandemia^a^**	
Não	60 (66,7)
Sim	30 (33,3)

^a^Variáveis com dados faltantes.

A adoção de protocolos para redução do risco da covid-19 pelo município foi relatada pela maioria dos cirurgiões-dentistas (67,1%). Já a adoção de alguma ferramenta da teleodontologia nesse período, foi relatada por 24,3% dos cirurgiões-dentistas. Dos que relataram o uso, a modalidade de teleodontologia mais utilizada foi a teleorientação (85,6%) (Figura 2). Entre os que declararam a implementação dessa tecnologia, apenas 33,3% acreditam que permanecerá sendo utilizada após a pandemia. 

**Figura 2:** Percentual das modalidades de teleodontologia utilizadas pelos cirurgiões-dentistas atuantes nas Unidades Básicas de Saúde do Brasil, ao final do segundo ano da pandemia de covid-19. Brasil, 2021 (n=90)

A Tabela 2 apresenta os resultados da análise univariada. Comparando as variáveis de exposição com o desfecho do estudo, observou-se associações positivas entre a implementação da teleodontologia e as Unidades Básicas de Saúde com cirurgiões-dentistas do sexo feminino (p-valor 0,049), que apresentavam profissionais com especialização em Saúde Pública ou Coletiva (p-valor 0,002), e se localizavam na região Sul do Brasil (p-valor<0,001) que as Unidades Básicas de Saúde que adotaram algum protocolo para reduzir o risco da covid-19 (p-valor<0,001).

**Tabela 2:** Comparação das variáveis de exposição do estudo com a implementação da teleodontologia na Atenção Primária a Saúde, no Brasil, ao final do segundo ano da pandemia da covid-19. Brasil, 2021 (n=416)

Variáveis	Implementação da teleodontologia		
	Não	Sim	
	n (%)	n (%)	p-valor
**Sexo** (n=416)			0,049
Masculino	88 (83,0)	18 (17,0) 83 (26,8)	
Feminino	227 (73,2)		
**Tempo de formado em odontologia** (anos) (n=415)^a^			0,429
0-5	89 (80,2) 67 (78,8) 48 (75,0) 38 (70,4) 70 (70,7)	22 (19,8) 18 (21,2) 16 (25,0) 16 (29,6) 29 (29,3)	
6-10			
11-15			
16-20			
≥21			
**Especialização em Saúde Pública ou Coletiva** (n=319)			0,002
Não	70 (86,4) 164 (68,9)	11 (13,6) 74 (31,1)	
Sim			
**Região brasileira da Unidade Básica de Saúde** (n=416)			<0,001
Sudeste	48 (57,8)	35 (42,2)	
Sul	168 (74,7)	57 (25,3)	
Centro-Oeste	16 (80,0)	4 (20,0)	
Norte e Nordeste	83 (94,3)	5 (5,7)	
**Adoção de protocolo pelo município da Unidade Básica de** **Saúde para reduzir risco de covid-19** (n=416)			<0,001
Não	119 (86,9)	18 (13,1)	
Sim	196 (70,3)	83 (29,7)	

^a^Variáveis com dados faltantes.

A Tabela 3 apresenta os resultados das análises multivariadas bruta e ajustada. Após a análise bruta, as variáveis de exposição que permaneceram associadas positivamente à implementação da teleodontologia foram: a região brasileira da Unidade Básica de Saúde, adoção de protocolo pelo município da Unidade Básica de Saúde para redução de risco da covid-19 e as Unidades Básicas de Saúde onde tinham cirurgiões-dentistas com especialização em Saúde Pública ou Coletiva. Na análise ajustada, essas mesmas variáveis também permaneceram associadas positivamente ao desfecho. A Unidade Básica de Saúde estar situada nas regiões Sul e Sudeste apresentou uma probabilidade 320,0% (RP 4,20; IC95% 1,71; 10,28) e 585,0% maior (RP 6,85; IC95% 2,74; 17,67), respectivamente, da implementação da teleodontologia se comparado com as regiões Norte e Nordeste. Além disso, aqueles cirurgiões-dentistas que relataram adoção de protocolo pelo município da Unidade Básica de Saúde para redução de risco da covid-19, tiveram uma probabilidade 59,0% maior de implementar a teleodontologia comparado com aqueles que não implementaram (RP 1,59; IC95% 1,01; 2,50). As Unidades Básicas de Saúde onde tinham cirurgiões-dentistas especialistas em Saúde Pública ou Coletiva apresentaram uma probabilidade 112,0% maior de implementar a teleodontologia comparado àquelas Unidades Básicas de Saúde que não tinham esses profissionais (RP 2,12; IC95% 1,20; 3,75). 

**Tabela 3:** Razões de prevalências (RP) e intervalos de confiança de 95% (IC95%) brutas e ajustadas da implementação da teleodontologia de acordo com as variáveis do estudo ao final do segundo ano da pandemia da covid-19. Brasil, 2021 (n=416)

Variáveis	Implementação da teleodontologia					
	Análise bruta			Análise ajustada		
	RP	(IC95%)	p-valor	RP	(IC95%)	p-valor
Sexo			0,052	-		
Masculino	1,00					
Feminino	1,57	(0,99; 2,49)				
**Tempo de formado em Odontologia** (**anos)a**			0,435	-		
0-5	1,00					
6-10	1,06	(0,61; 1,86)				
11-15	1,26	(0,71; 2,22)				
16-20	1,49	(0,85; 2,60)				
≥21	1,47	(0,91; 2,39)				
**Especialização em Saúde Pública ou Coletiva**			0,005			0,010
Não	1,00			1,00		
Sim	2,29	(1,27; 4,09)		2,12	(1,20; 3,75)	
**Região brasileira da Unidade Básica de Saúde**			0,001			0,001
Norte e Nordeste	1,00			1,00		
Centro-Oeste	3,52	(1,03; 11,96)		3,09	(0,87; 10,93)	
Sul	4,45	(1,84; 10,76)		4,20	(1,71; 10,28)	
Sudeste	7,42	(3,05; 18,05)		6,85	(2,74; 17,67)	
**Adoção de protocolo pelo município da Unidade Básica de Saúde para reduzir risco de covid-19**			0,001			0,046
Não	1,00			1,00		
Sim	2,26	(1,41; 3,61)		1,59	(1,01; 2,50)	

^a^Variáveis com dados faltantes.

## Discussão

A principal característica deste estudo foi identificar aspectos relativos à utilização da teleodontologia no Brasil durante a pandemia da covid-19. Seus resultados mostraram que, embora protocolos para redução de risco da covid-19 tenham sido implantados na maioria dos municípios onde atuavam os cirurgiões-dentistas envolvidos no estudo, ferramentas de teleodontologia foram utilizadas em apenas um quarto dos municípios, confirmando a hipótese do estudo. A teleorientação foi a modalidade da teleodontologia mais utilizada, segundo os participantes. Além disso, fatores como a localização da Unidade Básica de Saúde nas regiões Sul e Sudeste, e o fato de os cirurgiões-dentistas serem especialistas em Saúde Pública ou Coletiva favoreceram a utilização dessa ferramenta. Os autores deste estudo acreditam que os resultados obtidos, em um contexto de crise sanitária, ressaltam a importância da inclusão de modalidades de teleodontologia na rotina das equipes da Atenção Primária, promovendo maior prevenção e controle dos agravos relacionados à saúde bucal de toda população. Pelo conhecimento dos autores não há publicações sobre as ações da teleodontologia na Atenção Primária do Brasil durante a pandemia da covid-19. 

A adoção de protocolos municipais para redução do risco da covid-19 se manteve-se associada positivamente ao desfecho do estudo. É importante destacar que todas as medidas de biossegurança para o manejo clínico foram essenciais durante a pandemia, dado que os cirurgiões-dentistas mantinham contato direto com aerossóis que poderiam estar contaminados pelo vírus. Desta forma, os protocolos deveriam ser adotados desde a chegada do paciente até a sua saída do consultório, incluindo medidas para reduzir o tempo de espera e manejo clínico, visando minimizar o contato entre paciente e profissional (14). Um estudo que analisou os fatores associados à redução do número de atendimentos odontológicos realizados na Atenção Primária à Saúde no Brasil, no primeiro ano da pandemia, observou que 67,1% dos municípios adotaram protocolos para reduzir o risco de contaminação pelo vírus, e 29,7% implementaram algum tipo de triagem, (como aferição da temperatura, identificação de sinais e sintomas da covid-19, entre outras) na Unidade Básica de Saúde (15). A adoção de novos protocolos de biossegurança, incluindo a teletriagem, pode ter sido um fator importante para a implementação de ações de telemonitoramento e teleorientação observadas no presente estudo, principalmente para usuários que estavam em atendimento nas unidades de saúde antes do início da pandemia, pois a maioria dos municípios do Brasil durante primeiro ano da pandemia só realizaram atendimentos de urgência e emergência (3). 

As Unidades Básicas de Saúde com os cirurgiões-dentistas especialistas em Saúde Pública ou Coletiva implementaram mais ferramentas de teleodontologia comparados às Unidades Básicas de Saúde que não contavam com esses profissionais. Para um bom funcionamento da unidade de saúde e o desenvolvimento das ações de teleodontologia, é imprescindível que recursos humanos estejam capacitados para o uso das tecnologias. Profissionais da saúde especializados em Saúde Pública ou Coletiva são capacitados para reconhecer a importância e necessidade do aprimoramento contínuo dos serviços de saúde prestados à população. A presença de serviços bem estruturados e organizados, aliados a profissionais comprometidos como seu bom funcionamento, promove impactos positivos na saúde da população e aumenta a eficiência do sistema (16).

As Unidades Básicas de Saúde situadas nas regiões Sul e Sudeste implementaram mais ações de teleodontologia comparados as regiões Norte e Nordeste. A região Sul do Brasil ofereceu até agosto de 2024, o serviço de Telessaúde RS, uma iniciativa da Telemedicina do Ministério da Saúde em colaboração com a Universidade Federal do Rio Grande do Sul, desde 2007, com o objetivo de promover ações de teleconsulta e teleducação aos profissionais de Atenção Primária à Saúde (17). Este fato pode ter contribuído para o resultado encontrado no presente estudo, pois para a implementação de ações de teleodontologia, é necessário que exista uma infraestrutura técnica e de rede adequada, com conexão de internet apropriada, além da necessidade do treinamento da equipe. A adaptação dos profissionais ao sistema requer tempo para que o serviço possa ser utilizado de forma eficiente (18,19). 

O estudo coletou dados de todas as regiões do Brasil. No entanto, a amostra não foi representativa de todo país, pois a maior parte dos participantes do estudo era da região Sul do país (cerca de 50,0%), o que pode ser considerada uma limitação do estudo. Destaca-se, porém, que ocorreram esforços dos pesquisadores para contatar os cirurgiões-dentistas aptos a participar, utilizando e-mails e redes sociais. É importante ressaltar que, devido à pandemia, as pesquisas online eram o único meio de conhecer e entender como os serviços de saúde bucal na Atenção Primária à Saúde se organizaram para enfrentar a emergência de saúde pública. Um fator que pode contribuir para o fortalecimento das ações de teleodontologia no período pós-pandemia são os esforços da Secretaria de Informação e Saúde Digital do Ministério da Saúde na proposição de estratégias relacionadas à saúde digital para o período de 2020-2028, por meio da Rede Nacional de Dados em Saúde. Entre essas iniciativas, destaca-se a criação de uma plataforma digital de inovação, informação e serviços de saúde, com ações voltadas ao monitoramento e à avaliação da saúde digital em todo o Brasil (20). 

Por fim, considerando o objetivo deste estudo, pode-se afirmar que a adoção de protocolos para redução de risco da covid-19, a região brasileira da Unidade Básica de Saúde e a presença de um cirurgião-dentista com especialização em Saúde Pública ou Coletiva, foram associadas positivamente à implantação da teleodontologia. Além disso, observou-se um uso limitado das ferramentas de teleodontologia nas Unidades Básicas de Saúde do Brasil ao final do segundo ano da pandemia da covid-19. Esses resultados servem de alerta para que ações relacionadas à teleodontologia, especialmente aquelas voltadas ao monitoramento e à teleconsultoria, sejam implementadas de forma contínua e integrada à Política Nacional de Saúde Bucal, não apenas em momentos de crise sanitária, mas como uma estratégia permanente para a melhorar os indicadores de saúde bucal e, consequentemente, a qualidade de vida população.

## Data Availability

Após a publicação os dados estarão disponíveis sob demanda aos autores.
